# Case report: Muscular tuberculosis with lower-extremity muscular masses as the initial presentation: Clinicopathological analysis of two cases and review of the literature

**DOI:** 10.3389/fmed.2023.1106412

**Published:** 2023-03-14

**Authors:** Xiao-wei Zhu, Xing-hua Luan, Kai-li Jiang, Chao Zhang, Shi-hua Liu, Li Cao, Ping Zhong, Zhi-yan Liu

**Affiliations:** ^1^Department of Neurology, Suzhou Hospital of Anhui Medical University, Suzou, China; ^2^Department of Neurology, Shanghai Sixth People's Hospital Affiliated to Shanghai Jiao Tong University School of Medicine, Shanghai, China; ^3^Department of Pathology, Shanghai Sixth People's Hospital Affiliated to Shanghai Jiao Tong University School of Medicine, Shanghai, China

**Keywords:** tuberculosis, muscular tuberculosis, muscular mass, acid-fast bacilli stain, polymerase chain reaction

## Abstract

**Background:**

Tuberculosis (TB) is a threat to public health that mostly affects people in developing countries. TB presenting as a soft tissue mass is rare and is usually seen in patients with muscular tuberculosis (MT).

**Case presentation:**

In this study, we present the clinical, radiographic, and pathological features of two cases and retrospective evaluations of an additional 28 patients who were diagnosed with MT. More patients were men (60.9%) than women (39.1%), with a male-to-female ratio of 1.6:1. The average age among male and female patients was 38.9 and 30.1 years, respectively. MT usually presents with painful or painless muscular nodules on the lower limbs. Imaging findings, including ultrasound, CT, and MRI, can be used to identify lesions and sites for biopsy. The most typical histopathological feature of MT is granulomatous inflammation with caseous necrosis and epithelioid granulomata. Acid-fast bacilli stain and polymerase chain reaction (PCR) assays are helpful in identifying tubercle bacillus.

**Conclusion:**

We describe two MT cases with lower-extremity muscular masses as the initial presentation. The results suggest that muscle biopsy and pathological analysis remain necessary for diagnosis. Most of the patients could be cured with standard antituberculosis therapy.

## 1. Introduction

Tuberculosis (TB) is a public health threat throughout the world and ~10 million people contract TB every year. The bacteria usually attack the lungs, butcan also attack other parts of the body, such as the kidney, spine, and brain. Extrapulmonary cases of TB are distributed among the lymph nodes (47%), pleural cavity (30%), abdomen (10%), bones and joints (8%), CNS (2%), and other sites (3%). The least frequent location of TB is intramuscular (1, 2). TB presenting as a soft tissue mass is rare and is usually seen in patients with muscular tuberculosis (MT). MT mainly presents in the form of muscle masses at a single site or multiple sites with swelling, weakness, pain, or painlessness (3, 4). The rarity of this condition often leads to failure to consider MT in the differential diagnosis, resulting in delayed therapy.

Here, we report two MT cases with muscular nodules on the lower limbs as the initial presentation. Furthermore, we present a retrospective evaluation of an additional 28 patients diagnosed with typical MT, drawn from 27 articles in the PubMed database published between 2000 and 2022, with the aim of determining the clinicopathological characteristics of MT and establishing definitive differential diagnosis criteria.

## 2. Case presentation

### 2.1. Case 1

A 66-year-old woman presented with a 1-month history of a painful isolated mass on the inside of the left calf. She also complained of bilateral numbness in her toes. A history of contact with patients with suspected TB could be traced: her father had had pulmonary TB 10 years previously, and her daughter had had tuberculous pleurisy 7 years previously. She was in good general condition and did not complain of systemic symptoms such as night sweats, weight loss, anorexia, fatigue, or intermittent fever. Physical examination revealed a tender mass with irregular indistinct borders in her left calf. Movement was severely restricted by knee pain. She had a slight decrease in sensation and swelling of the lower extremities. The local temperature was not elevated, and the skin over the mass was normal. No obvious mass was palpable on the right leg. Further clinical examinations, including hemogram, erythrocyte sedimentation rate (ESR), procalcitonin test (PCT), liver and renal function tests, antinuclear antibody (ANA), antineutrophil cytoplasmic antibodies (ANCA), fungal G-test, and tumor marker levels, returned results within normal limits. Serological rheumatoid factor (RF) was elevated to 33.8 iu/ml (reference range: up to 15 iu/ml), serological high sensitivity C-reactive protein (CRP) waselevated to 7.48 mg/L (reference range: up to 3 mg/L), and antistreptolysin O (ASO) was positive. Tuberculin antibody IgG, IgM, γ-interferon release test, purified protein derivative (PPD) skin test, acid-fast bacilli smear, and tissue culture of sputum were negative. Electromyography (EMG) indicated reduced motor and sensory nerve conduction velocity in the lower limbs (MCV: 25.8–33.1 m/s, SCV: 0–34.3 m/s), accompanied by decreased compound motor action potential (CMAP: 1.6–12.1 mV) and sensory nerve action potential amplitude (SNAP: 0–2.1 uV). Distal motor latency and *F* wave were normal. The duration and amplitude of motor unit potential (MUP) were increased, without spontaneous activity, indicating extensive chronic neurogenic lesions.

**Figure 1:**
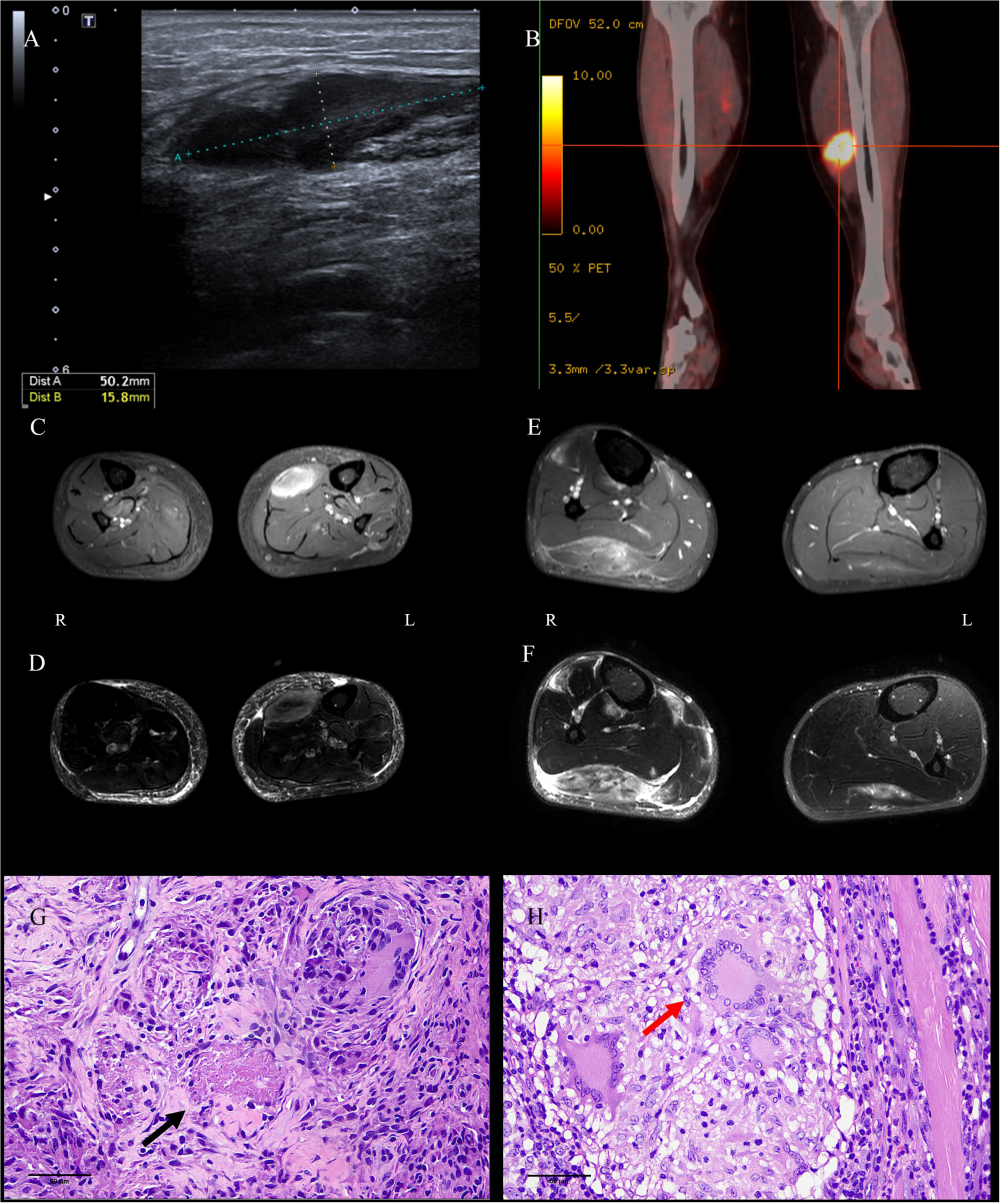
Images and pathological findings of the muscle masses. The lesion in the left soleus muscle of P1 was found to be hypoechoic on ultrasound **(A)**, hypermetabolic on PETCT **(B)**, enhanced on axial enhanced T1WI-TES **(C)**, and light-hyperintense on T2W-SPAIR **(D)**. Several well-formed epithelioid granulomas centered by caseous necrosis were observed [**(G)**, HE, bar = 50 μm]. The lesion in the right gastrocnemius muscle of P2 was identified *via* enhancement on axial enhanced T1WI-TES **(E)** and hyperintensity on T2W-SPAIR **(F)**. Three Langerhans cells in granulomas were noticed in the sections [**(H)**, HE, bar = 50 μm]. P, patient; PETCT, positron emission tomography–computed tomography; T1W-TSE, T1-weighted turbo spin echo; T2W-SPAIR, T2-weighted spectral presaturation attenuated inversion recovery.

Chest computed tomography (CT) of case 1 revealed scattered bilateral inflammatory changes in the inferior lobes of both lungs and multiple nodular thickening on both sides of the pleura. Bilateral ultrasound of the calves suggested a 50 × 16-mm hypoechoic lesion in the left soleus muscle (Figure 1A) and a 43 × 6-mm hypoechoic lesion in the medial head of the gastrocnemius muscle. Additionally, 18 F-fluorodeoxyglucose positron emission tomography–computed tomography (18 F-FDG-PETCT) revealed multiple hypermetabolic lesions, which involved the left soleus muscle, the left musculus fibularis longus, the right soleus muscle, and the tibialis anterior muscle. The largest lesion was located on the middle of the left soleus muscle measuring 33.8 × 20 × 42 mm, and the maximum standardized uptake value (SUVmax) was 15.9 (Figure 1B). Magnetic resonance imaging (MRI) examination showed abnormal mass signals involving the inner front edge of the gastrocnemius muscle. The lesion was enhanced by gadolinium on the T1-weighted turbo spin-echo (T1W-TSE) image, and T2-weighted spectral presaturation attenuated inversion recovery (T2W-SPAIR) showed a hyperintense mass. There was a similar lesion in the gastrocnemius muscle of the right leg (Figures 1C, D). Pelvis and lumbar spine MRI showed mild lumbar disk herniation, with no other abnormal findings.

A muscle biopsy of the left gastrocnemius was performed in case 1; the result showed multiple nodular epithelioid granulomas embedded among the muscle fibers. Caseous necrosis was found under the microscope (Figure 1G). Microscopic examination for acid-fast bacilli stain and cytokeratin (AE1/AE3) was completely negative. CD3, CD31, CD34, desmin, Kp1, and PGM1 staining were weakly positive. The Ki67 labeling index was low (10%). PCR for the mycobacterium tuberculosis complex in blood, urine, sputum, and paraffin-embedded samples from case 1 returned negative results; however, the result was positive in frozen sections of muscles. A diagnosis of MT was considered in case 1, despite the fact that all the tests used for diagnosis of TB were negative, with the exceptions of PCR and histopathology (5, 6).

### 2.2. Case 2

A 50-year-old woman presented with a mass in the right calf for 4 months. There were no symptoms of cough or weight loss, and no history of intramuscular injection at the site concerned or of any contact with TB. Physical examination revealed an indurated nodule with no tenderness or elevation of local temperature, about 8 × 5 cm in size. The borders of mass were not well-demarcated. There was no abnormality of distal vascularity or sensation, and no limitation of movement. Laboratory tests revealed a normal blood count, and liver function tests, ASO titers, and renal functions were not deranged. An extensive laboratory workup, including ANA, ANCA, CRP, tumor marker levels, thyroid function tests, and inflammatory markers, returned results within normal limits. The outcomes of several infection tests for syphilis, hepatitis B, hepatitis C, and HIV were negative.

Ultrasound of the right calf was performed in case 2 and suggested a hypoechoic lesion. The patient in case 2 also underwent musculoskeletal MRI examination, and the result showed similar lesions to case 1 in the soleus muscle and medial posterior part of the right tibia and fibula (Figures 1E, F). According to radiological imaging findings, both patients had other painless muscular nodules at multiple sites throughout their entire bodies.

Resections and histopathologic observations of lesions were performed in case 2. Granulomatous inflammation, mainly composed of epithelioid histiocytes and Langerhans giant cells, was observed under the microscope (Figure 1H). Histopathologic examination showed the presence of non-necrotizing epithelioid granulomas compatible with TB. Immunohistochemistry against CD4/CD8, MHC-I, and MAC (C5b9) was negative. Moreover, the acid-fast bacilli stain was negative. PCR for TB was positive in case 2 in paraffin-embedded and frozen sections of muscles. A diagnosis of MT was considered in case 2, despite the lack of detectable focus of tubercular infection except in the PCR and histopathology results (5, 6).

Many differential diagnoses were considered in both cases, including deep vein thrombosis, a soft tissue tumor, or a pyogenic abscess. Based on the diagnosis of MT, the patients in cases 1 and 2 were both prescribed standard oral antituberculosis treatment with four drugs, namely, isoniazid, rifampicin, ethambutol, and pyrazinamide, for 2 months. After treatment, the patients' symptoms were relieved, and the nodules in the calves appeared to decrease in size. Further courses of isoniazid and rifampicin were administered. At the time of writing, the patients are undergoing follow-up.

## 3. Discussion

In recent years, as the incidence of pulmonary TB has rebounded, extrapulmonary TB has become a common disease (7). MT without bony involvement is still a rare presentation, as it has been since it was first reported in 1886. Since 2000, only 28 cases of MT have been reported in the published literature (3, 6, 8–32). Combining these with the two new cases reported here, we reviewed all 30 cases; the clinical profiles are summarized in Table 1.

**Table 1:** Clinical profiles of reported cases of MT.

**Year**	**Case no**.	**Age/sex**	**Course of disease**	**Involved body parts main clinical manifestations**	**Diagnosis methods**	**References**
2001	1	23/M	A few weeks	Forearm, thigh, calf	Acid-fast bacilli smear, tissue culture of lesion, and biopsy	Haq et al. (8)
				Mass, pain, weakness		
2001	2	24/M	5 weeks	Left psoas	PPD skin test, acid-fast bacilli smear, and tissue culture of lesion	Franco-Paredes et al. (10)
				An enlarging soft tissue mass, pain		
2001	3	49/M	1.5 months	Gluteus	Tissue culture of lesion and biopsy	Ergin et al. (9)
				Mass, left leg pain, fever		
2002	4	9/F	8 weeks	Pectorales, forehead	Tissue culture of lesion	Morris et al. (11)
				Pain; tender, fluctuant swellings		
2002	5	11/M	2 months	Biceps brachii	Biopsy	Trikha et al. (12)
				Swelling, non-tender		
2003	6	9/F	2 months	Right calf	Biopsy	Bakshi et al. (13)
				Swelling, non-tender		
2005	7	53/M	Chronic occult	Pectorales	Biopsy	Winzer et al. (14)
				A fixed tender mass		
2006	8	30/F	4 months	Thigh	Tissue culture of lesion and biopsy	Trikha et al. (15)
				Swelling, pain		
2009	9	23/M	3 months	Right forearm	PPD skin test and acid-fast bacilli smear	Narang (17)
				Enlarging soft tissue masses, non-tender		
2009	10	32/F	10 weeks	Left masseter	PCR for mycobacterium tuberculosis	Mascarenhas et al. (16)
				Swelling, tender		
2010	11	55/M	3 months	Right forearm, both thighs	Acid-fast bacilli smear and biopsy	Huang et al. (18)
				Swelling, tenderness, and localized heat		
2011	12	71/M	6 months	Right thigh	Tissue culture of lesion and biopsy	Perez-Alonso et al. (19)
				Ill-defined mass, symptomatic, tender		
2012	13	15/M	NA	Left thigh	Tissue culture of lesion and biopsy	Arora et al. (20)
				Swelling, tender		
2013	14	25/M	3 months	Right gluteus	PPD skin test, acid-fast bacilli smear, and tissue culture of lesion	Elshafie et al. (21)
				Enlarging swelling		
2013	15	11/F	4 months	Right thigh, right calf, and left arm	PPD skin test, tissue culture of lesion, and biopsy	Neogi et al. (22)
				Swelling, painless		
2013	16	37/F	2 months	Right upper arm, biceps brachii muscle	PPD skin test, acid-fast bacilli smear, tissue culture of lesion, and biopsy	Sokucu et al. (23)
				Mass, pain, swelling		
2015	17	25/M	1 months	Right triceps	PPD skin test, tissue culture of lesion, PCR for mycobacterium tuberculosis, and biopsy	Meena et al. (24)
				Hard, fixed swelling, slight tenderness		
2015	18	9/F	3 months	Forearm, right calf	Biopsy	Dhakal et al. (6)
				Swelling, painless		
2016	19	38/F	2 months	Dorsal muscles	Tissue culture of lesion and biopsy	Dabó et al. (25)
				Pain and progressively increasing swelling		
2016	20	24/M	2 years	Pectorales	PPD skin test, acid-fast bacilli smear, tissue culture of lesion, and biopsy	Grigorakos et al. (26)
				Swelling and pain		
2016	21	38/M	2 years	Pectorales	PPD skin test, acid-fast bacilli smear, tissue culture of lesion, and biopsy	Grigorakos et al. (26)
				Swelling and pain		
2016	22	83/M	NA	Anterior tibia muscle of left calf	Biopsy	Kotecha et al. (27)
				Swelling and tenderness		
2017	23	33/F	2 weeks	Right calf	Acid-fast bacilli smear, tissue culture of lesion, and biopsy	Al-Khazraji et al. (29)
				Pain, weakness, slight swelling, and redness		
2017	24	23/F	NA	Left thigh	PPD skin test, PCR for mycobacterium tuberculosis, and biopsy	Alaya and Osman (28)
				Swelling, pain		
2019	25	49/M	9 months	Both thighs, calves	PCR for mycobacterium tuberculosis, tissue culture of lesion, and biopsy	Zeng et al. (3)
				Mass, swelling, pain		
2020	26	45/F	3 months	Flexor carpi radialis	Biopsy	Fahad et al. (30)
				Swelling, pain		
2022	27	38/F	4 months	Psoas	Tissue culture of lesion and biopsy	Mohandes et al. (31)
				Pain		
2022	28	57/M	NA	Thigh	PCR for mycobacterium tuberculosis and biopsy	Zhang et al. (32)
				Swelling		
2022	29	66/F	1 month	Both calves	Biopsy	
					Mass, pain, bilateral numbness in toes	
2022	30	50/F	4 months	Both calves	PCR for mycobacterium tuberculosis and biopsy	
				Mass, painless		

F, female; M, male; NA, not available.

MT occurred more often in men (60.9%) than in women (39.1%), with a male-to-female ratio of 1.6:1. The average age among male and female patients was 38.9 and 30.1 years, respectively (range: 9–83 years). All patients presented with chronic occult onset, ranging from 2 weeks to 2 years, and symptoms gradually became more aggravated. There were 11 cases of patients who presented with masses as the main clinical manifestation, and the other 19 patients presented with swelling. Among the 11 cases, seven were recorded as having nodular mass in the lower extremities (3, 8, 13, 19). Pain (20/30) was the most commonly presenting clinical feature. The thigh and calf (9/30, respectively) were the most common sites of involvement. This symptom and location were distinctive features for MT and should be included in the differential diagnostic criteria for other muscular nodular diseases. In five cases (including case 1 in the present report), the patient had suffered from pulmonary TB or had a history of contact with patients with suspected TB (3, 14, 21, 29). At present, it is generally agreed that hematogenous dissemination plays an important role in MT, although in some cases it was transmitted through an infected needle (3, 6, 15, 20, 21). Skeletal muscles were usually spared by TB because these are a poor host for mycobacterium tuberculosis (30). In consideration of MT occurring as a secondary infection, patients in four cases had been treated with steroids, and long-term chronic pulmonary inflammation or decreased immunity may therefore be a potential risk factor for MT (3, 9, 18, 27, 29). The clinical symptom in case 1 was painful isolated masses in the lower limbs, and EMG suggested potential generalized involvement of peripheral nerves with axonal damage. We speculate that MT may affect the peripheral nerves, which has not been reported previously. Unfortunately, further biopsy of the sural nerve was not performed to prevent secondary infection.

**Figure 2:**
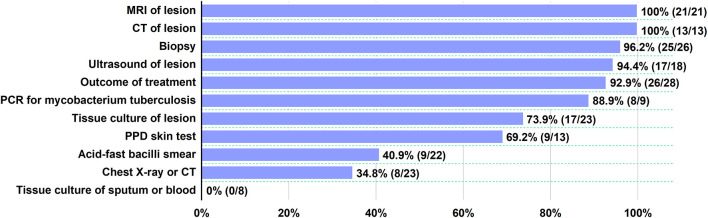
Diagnostic tests for TB disease. For each test, the proportion of positive patients is indicated.

Diagnostic tests for TB disease had been performed on all 30 patients, including PPD skin test (9/13), acid-fast bacilli smear (9/22), tissue culture of sputum or blood (0/8), tissue culture of the lesion (17/23), PCR for MT (8/9), chest X-ray or CT (8/23), ultrasound (17/18), CT (13/13), MRI (21/21), and biopsy (25/26) of the lesion (Figure 2) (3, 6, 18–32). Pathological information was provided for 26 patients. The most common histological features of MT included granulomatous inflammation, caseous necrosis, and epithelioid granulomata. The TB test could be partially negative, which does not rule out TB infection (26). Among the available modalities, serological tests and PPD skin tests were not recommended in the diagnosis of MT or the initiation of antituberculosis treatment. Bacterial culture of the lesion helps to demonstrate the etiological organism, but culturing was not sensitive in sputum or blood. It should be highlighted that in previous studies, most of the patients were diagnosed as MT based on muscle biopsy, even though some of these patients (5/26) did not have a positive result associated with TB infection (12, 30). TB pathognomonic lesions could be found at any site of TB infection. This was a characteristic granulomatous inflammatory reaction against mycobacterium tuberculosis bacilli from the host's cell-mediated immunity (33). The diagnosis of MT mainly depended on biopsy and bacteriological culture traditionally. PCR was reported recently to be a useful method to determine TB (5). In our cases, TB was not detected by acid-fast bacilli stain, but PCR for TB proved positive in preserved frozen sections of muscles. However, PCR was negative in the blood, urine, and sputum in case 1, probably because the trace amounts of TB DNA were not detected. Sometimes, DNA-related tests using paraffin-embedded samples showed inconsistent results. This indicates that PCR is more sensitive than acid-fast bacilli stain, especially at the site of infection. This is consistent with earlier findings (3, 32).

Imaging findings, including ultrasound, CT, and MRI, could identify lesions and sites for biopsy (28, 34). The evaluation should begin with ultrasonography. The tissue masses of the soft parts had a hypoechogenic appearance in most cases. Sonography allowed for differentiation between solid and liquid lesions, and some soft tissue tumors, hematomas, and cystic diseases could be distinguished (35). However, ultrasound was a very sensitive but non-specific examination. In general, a soft tissue mass of unknown character in the lower extremity requires further workup *via* MRI, because this may confirm the size and nature of the mass more clearly than ultrasound, particularly in indicated tissue swelling and deeper tissue damage (36).

The differential diagnosis list for MT is broad, especially in the case of some patients presenting clinically with muscle nodules. The most common differentials include schwannomas, lipoma, liposarcoma, ganglionic cyst, focal myositis, and hydatid cyst of the muscle. Because of a suspicion of malignancy, exploration was undertaken and an open biopsy was conducted on the nodule in two patients. Focal myositis could also present with similar clinical and imaging presentation, but the pathological features such as inflammatory infiltration and muscle fiber atrophy, necrosis, and regeneration were not obvious in the muscle pathology of these two patients. In fact, during diagnosis, oncological vigilance should be maintained, because MT presentation is similar to that of some patients with tumors.

The main treatments were standard antituberculosis therapies and lesion removal. Observations of therapeutic effects and prognostic data were available in 28 cases (including ours). Almost all diagnosed patients were treated with standardized application of TB drugs, and most had good prognoses (26/28) (3, 6, 9–13, 24–32). Only two patients died of another fatal disease during therapy (cerebrovascular hemorrhage; profound shock and multiorgan failure) (9, 18).

In conclusion, we have described two patients who presented with muscular nodules on the lower limbs as the initial manifestation of tuberculosis and presented the corresponding clinical, imaging, and pathological features. Although MT is a rare entity, it should be considered in a differential diagnosis when a patient presents with single or multiple intramuscular masses, especially in tubercular endemic areas. PCR is a sensitive and useful method to confirm MT.

## Data availability statement

The original contributions presented in the study are included in the article/supplementary material, further inquiries can be directed to the corresponding authors.

## Ethics statement

The studies involving human participants were reviewed and approved by the Ethics Committee of Shanghai Sixth People's Hospital Affiliated to Shanghai Jiao Tong University School of Medicine (Approval No: 2021-219). The patients/participants provided their written informed consent to participate in this study. Written informed consent was obtained from the individual(s) for the publication of any potentially identifiable images or data included in this article.

## Author contributions

X-wZ: data acquisition, data analysis, interpretation of data, and drafting of the manuscript for intellectual content. X-hL: data acquisition, analysis of data, and revision of the manuscript for intellectual content. K-lJ, CZ, S-hL, and LC: data acquisition. PZ and Z-yL: funding, study design, conceptualization, data analysis, interpretation of data, manuscript revision, and supervision. All authors contributed to the article and approved the submitted version.
